# Frequency and Demographic Analysis of Odontogenic Tumors in Three Tertiary Institutions: An 11-Year Retrospective Study

**DOI:** 10.3390/diagnostics14090910

**Published:** 2024-04-26

**Authors:** Asma Almazyad, Mohammed Alamro, Nasser Almadan, Marzouq Almutairi, Turki S. AlQuwayz

**Affiliations:** 1 Maxillofacial Surgery and Diagnostic Sciences Department, College of Dentistry, King Saud bin Abdulaziz University for Health Sciences, Riyadh 11481, Saudi Arabia; alamro679@ksau-hs.edu.sa (M.A.); marzouqalmutairy@gmail.com (M.A.); 2 King Abdullah International Medical Research Center, Riyadh 11481, Saudi Arabia; 3 Department of Pathology and Laboratory Medicine, King Abdulaziz Medical City, Ministry of National Guard Health Affair, Riyadh 11426, Saudi Arabia; 4 Prince Sultan Military Medical Center, Riyadh 11159, Saudi Arabia; nmalmadan@gmail.com (N.A.); turkisaad1@hotmail.com (T.S.A.); 5 Dental Specialist Center, Hafar AlBaten 39953, Saudi Arabia

**Keywords:** ameloblastoma, biopsy, odontoma, odontogenic tumors, odontogenic myxoma, tertiary hospitals

## Abstract

Odontogenic tumors (OTs) are distinct conditions that develop in the jawbones, exhibiting diverse histopathological features and variable clinical behaviors. Unfortunately, the literature on this subject in Saudi Arabia remains sparse, indicating a pressing need for more comprehensive data concerning the frequency, demographics, treatment modalities, and outcomes of OTs. Objectives: The study aims to evaluate the frequency, demographic features, treatment, and outcomes of OTs across three tertiary medical centers. Methods and Material: OT cases were identified in King Abdulaziz Medical City (KAMC), King Fahad Medical City (KFMC), and Prince Sultan Military Medical City (PSMMC) from January 2010 to December 2021. Results: Ninety-two OT cases were identified from the anatomical pathology laboratories of three tertiary hospitals. KFMC contributed the highest number of cases (43.5%), followed by KAMC (30.4%) and PSMMC (26.1%). The median age of OT patients was 29 years (range: 5–83), with males representing more than half of the patients (56.5%). The mandible was the most frequent site of OT occurrence (72.5%), with ameloblastoma being the predominant OT (63.0%), followed by odontoma (19.5%). Among the treatment modalities, bone resection was employed the most (51.0%), followed by enucleation (25.6%). Notably, 11.5% of OT cases with available follow-up data exhibited recurrence, with ameloblastoma accounting for eight recurrent cases. Conclusions: Although OTs are relatively common in the jaws, they are rare in anatomical pathology laboratories and the general population. This study contributes valuable insights into the epidemiology characteristics, treatment trends, and recurrence rates of OTs in Saudi Arabia.

## 1. Introduction

Odontogenic tumors (OTs) constitute a significant category of lesions primarily occurring in the jawbone, with occasional occurrence in the gingiva. These tumors originate from abnormal odontogenesis processes or the proliferation of odontogenic epithelial and odontogenic ectomesenchyme remnants [1,2]. Histologically, OTs display features reminiscent of various odontogenic tissues and development stages. The World Health Organization (WHO) categorizes OTs based on tissue type, with the latest 2022 classification featuring six purely epithelial, four purely mesenchymal, and four mixed tumors. Many OTs have clinical or histological subtypes reviewed and summarized in Table 1 [2]. While the existing literature and systematic reviews offer insight into the frequency and demographics of OTs, with reported rates ranging from 1.8% to 9.6% across various geographic locations [3,4,5,6,7], a notable research gap exists regarding OTs in Saudi Arabia. Only three published papers [8,9,10], including a study from Riyadh, Saudi Arabia, by Alsheddi et al. [10], reviewed 188 cases in a single oral pathology laboratory over twenty-six years. Among the commonly reported OTs, ameloblastoma (AM) was the most prevalent, followed by odontoma (OD), while less common tumors included cementoblastoma (CB) and central odontogenic fibroma (OF) [11]. A primordial odontogenic tumor (POT), a rare OT, was not included in most OT series as it was only described in 2014 and subsequently incorporated by the WHO classification in 2017 [12].

The existing literature primarily focuses on demographics, clinical characteristics, and histological examination, lacking substantial information on clinical behavior and treatment options. In response to this gap, our study aims to report OT frequency, demographic data, treatment modalities, and follow-up information, drawing from three tertiary hospitals in Riyadh, Saudi Arabia.

## 2. Methods and Materials

This retrospective study aimed to analyze the frequency, demographic data, and biological features of patients diagnosed with OTs at three tertiary medical hospitals in Riyadh, Saudi Arabia, King Abdulaziz Medical City (KAMC), King Fahad Medical City (KFMC), and Prince Sultan Military Medical City (PSMMC) from January 2010 to December 2021. The study adhered to the principles of the Declaration of Helsinki. The authors obtained institutional review board approval from the King Abdullah International Medical Research Center (IRB# NRC21R/222/06) and King Fahad Medical City (IRB# 00010471).

**Figure 1:**
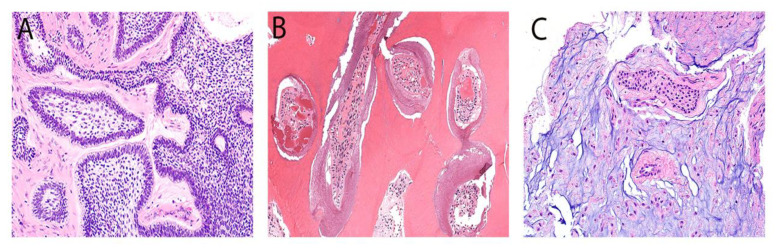
Representative histological images of the most common OTs: AM (**A**), OD (**B**), and OM (**C**). (**A**) AM displays multiple follicular islands with central stellate reticulum cells and peripheral basophilic columnar cells with reverse polarity. (**B**) OD exhibits multiple hard dental structures, such as the dentinal tubules and enamel matrix, with a fish-scale appearance. (**C**) OM shows multiple odontogenic rests with peripheral hyalinization and a paracellular myoxid background.

**Figure 2:**
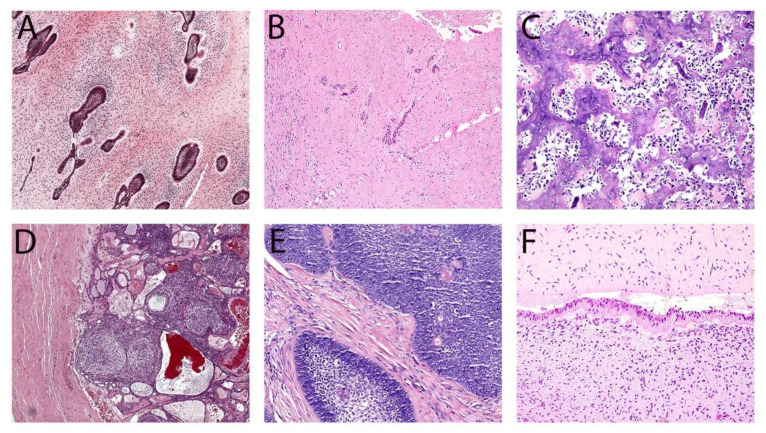
Representative histological images of other OTs seen in the current series: AF (**A**), OF (**B**), CB (**C**), AOT (**D**), AM carcinoma (**E**), and POT (**F**). (**A**) AF shows multiple ameloblastic islands within primitive ectomesenchymal stroma. (**B**) OF exhibits diffuse fibrotic stroma with scattered odontogenic epithelial rests. (**C**) CB shows hard tissue deposits similar to the cementum lined by multiple layers of cementoblasts and multinucleated giant cells. (**D**) AOT exhibits a fibrous capsule with nodular growth of epithelial and spindle cells with a whirling pattern and duct formation. (**E**) AM carcinoma displays ameloblastic islands with high cellularity, hyperchromasia, and keratin pearls. (**F**) POT consists of primitive ectomesenchymal tissue lined by a single layer of columnar epithelium with reverse nuclear polarity.

All relevant data, including patient age, sex, tumor site, treatment modalities, and follow-up information, were extrapolated from medical records. Hematoxylin and eosin-stained slides from all cases were retrieved and cross-examined by a certified oral pathologist (AA) to confirm the histopathologic diagnosis based on the 2022 WHO classification of odontogenic and maxillofacial bone tumors. In cases of uncertainty, another oral pathologist (NM) conducted a secondary review to reach a consensus. Figure 1 and Figure 2 show representative histopathological photomicrographs of each observed OT in this series.

The inclusion criteria are as follows:

1. Tumors with histopathological features compatible with an OT diagnosis.
2. Patients with available demographic and clinical information, pathological reports, and histological slides.

The exclusion criteria are as follows:

1. Patients with incomplete demographic and clinical data.
2. Patients with missing histological slides.

Descriptive analysis of patient age, sex, site, OT type, treatment, and follow-up information was performed using STATA 14.2 software (StataCorp.), College Station, TX, USA. The correlation between age, gender, location, and OT type among different tertiary hospitals was assessed using the chi-square test for categorical data and the Kruskal–Wallis test for continuous data since the data failed the normal distribution test. A *p*-value less than 0.05 was considered statistically significant. Graphs were generated using STATA 14.2 software (StataCorp.), College Station, TX, USA.

## 3. Results

**Figure 3:**
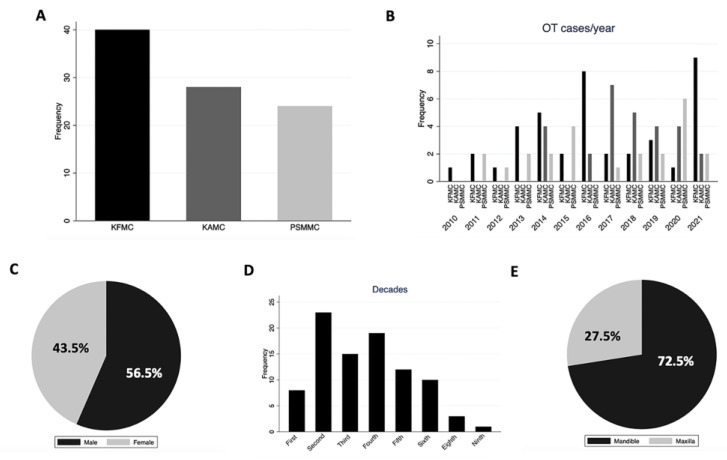
(**A**) OT distribution in each center. (**B**) OTs are distributed in each center per year. KFMC had the highest frequency in 2016 and 2021. (**C**) Gender distribution of OTs. (**D**) Age distribution of OTs. (**E**) Location distribution of OTs.

**Figure 4:**
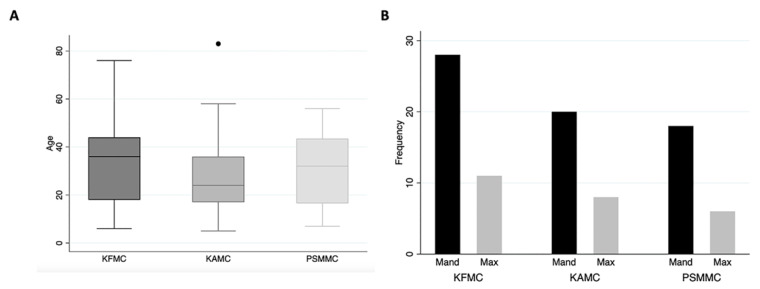
(**A**) The mean age of OTs is similar in all hospitals, with a *p*-value of 0.40. (**B**) Site distribution of OTs in each hospital, with a *p*-value of 0.89.

In 11 years, ninety-two cases of OTs were identified in the archives of three different tertiary hospitals in Riyadh, Saudi Arabia. The retrospective analysis revealed that forty (43.5%) and twenty-eight (30.4%) tumors originated from KFMC and KAMC, respectively. Additionally, twenty-four tumors were retrieved from PSMMC (Figure 3A). The distribution of cases per year in each hospital is illustrated in Figure 3B, highlighting peak occurrence in 2014, 2016, and 2021. Overall, OTs affected 52 (56.5%) males and 40 (43.5%) females, resulting in a male-to-female ratio of 1.3:1. Notably, KAMC exhibited a higher ratio of 1.54:1. At the same time, PSMMC showed an equal distribution between genders (Figure 3C and Table 2). The median age of the patients was 29 years, with most OT cases diagnosed in the second-to-fourth decades, constituting 63.0% (Figure 3D). No significant age differences were observed among the three hospitals, with a *p*-value of 0.40 (Table 2 and Figure 4A). Mandible involvement was predominant, accounting for almost two-thirds (72.5%) of the OT cases, with consistent site distribution across all hospitals (Figure 3E and Figure 4B and Table 2).

**Figure 5:**
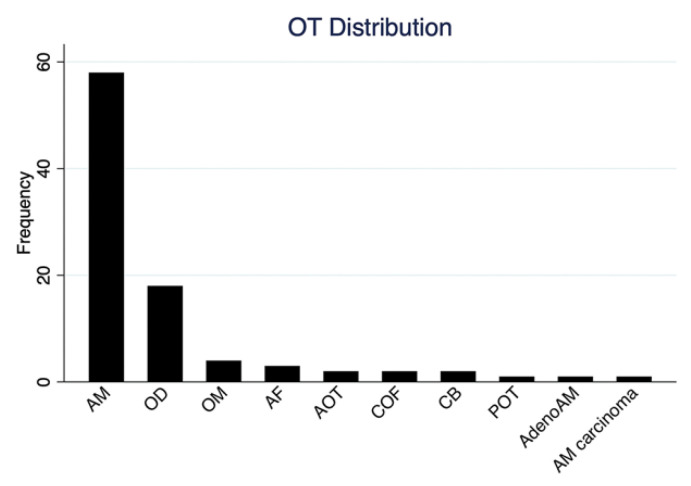
OT frequency in the three tertiary hospitals.

AM represented most cases across all centers, accounting for 63.0% (58 cases), making it the most prevalent OT. OD was the second most common OT, constituting 19.5% of the cases (Figure 5). Other OTs included four cases of OM (4.3%) and three cases of AF (3.3%). Only two COF, two AOT, and two CB cases were identified in the cohort. Additionally, there was one POT case from KFMC. PSMMC reported the sole case of malignant OT within the cohort. The distribution of OTs differed significantly among the hospitals, with a *p*-value of 0.01. At KFMC and PSMMC, AM predominated, while KAMC exhibited a more balanced distribution between AM and OD. KFMC also had the most OM and OF cases (Table 3).

Table 4 provides an overview of the clinicopathological characteristics of OTs. Among 82 patients with available treatment information, resection was the most common treatment modality (51.0%), followed by enucleation (25.6%), as outlined in Table 5. Additionally, follow-up data for 73 cases revealed recurrence in 11 (15.1%) cases (Table 6).

**Figure 6:**
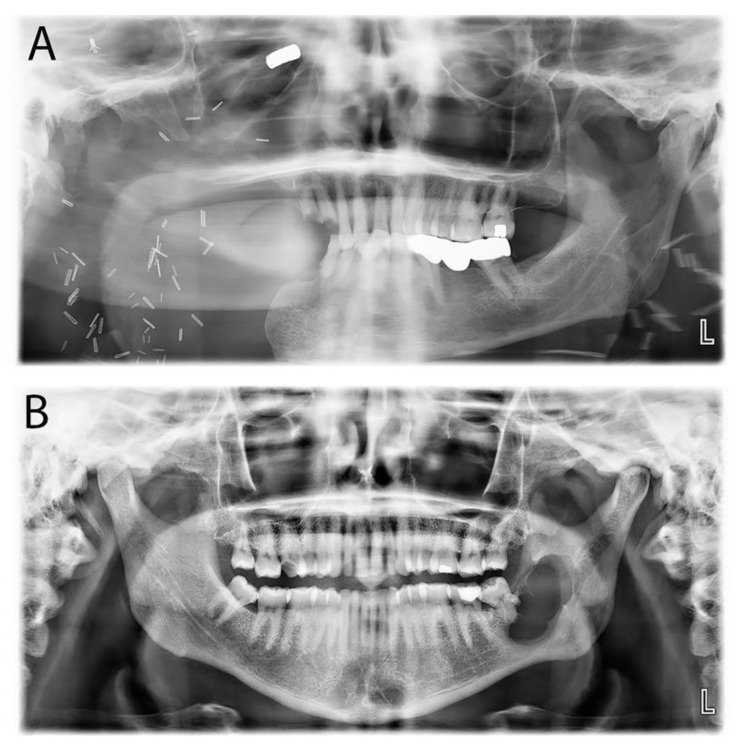
Examples of radiographic presentations of recurrent ameloblastoma. (**A**) Recurrent ameloblastoma on the right posterior mandible post-resection. (**B**) Recurrent ameloblastoma on the right posterior mandible post-excision.

Fifty-eight cases (63.0%) of AMs were identified, with a median age of 36, of which 34 (58.6%) were males. The mandible was the most common location (81.0%). Primary treatment modalities for AM included resection (66.7%) and excision (25.0%) (Table 5). Follow-up data for fifty cases revealed that only eight patients showed recurrence (Figure 6). Recurrence rates did not show any notable variation across the various treatment choices. Most AMs were of the conventional clinical subtype, with only four unicystic cases. No peripheral AM cases were reported. The main clinicopathological difference between conventional and unicystic AM was the median age and gender distribution, with unicystic AM tending to occur in females in their second decade.

There were 18 (19.5%) cases of OD with a median age of 20, of which 10 (55.6%) were males. Similar to AM, ODs showed almost equal site predilection with a ratio of 0.88:1 for the mandible-to-maxilla (Table 4). For OD, the primary treatment modality was enucleation (70.6%), with only one case treated with resection (Table 5). Seventeen patients with OD were followed up after treatment, and only one patient showed recurrence (Table 6).

OM ranked as the third most common OT, with only four cases (4.4%) reported in the cohort. The median age of patients was 31, and 3 (75%) were male. There was an equal preference for location between the mandible and maxilla (Table 4). Additionally, two-thirds of the cases were treated with resection. All cases were followed-up, and the case treated with excision showed recurrence (Table 5 and Table 6). The limited number of other OTs precluded extracting meaningful insight regarding clinicopathological information and treatment options.

Interestingly, the series had one case of AdenoAM in a 14-year-old female with a history of familial adenomatoid polyposis who died after six years due to encephalopathy. It is worth noting that there was only one case of malignant OT, histologically diagnosed as ameloblastic carcinoma in a 31-year-old male in the mandible, and it was resected. However, this patient was lost to follow-up.

## 4. Discussion

The present study marks a significant advancement in understanding OTs within Riyadh, Saudi Arabia. This retrospective multicenter series draws data from three leading tertiary hospitals in Riyadh, Saudi Arabia. It is the second-largest series of OT cases documented within the Kingdom and the wider Gulf region. Traditionally, studies on the frequency and prevalence of OTs have been confined to single institutions, potentially skewing the perception of their actual occurrence within a given geographic area. In addition, our prior examination of odontogenic cysts from these same hospitals yielded 372 cases, shedding light on their prevalence in this region [13]. Our findings reveal a notable contrast as follows: odontogenic cysts (OCs) are observed to be four times more frequent than OTs in Riyadh, Saudi Arabia, which is a significantly higher ratio than previously reported figures (OCs were reported to be 2.2 times more common than OTs) [11]. One possible explanation for this notable difference is that the previously reported ratio included the odontogenic keratocyst as an OT, which has since been reclassified as a cyst. This highlights the importance of comprehensive, multicenter studies in accurately capturing the epidemiological landscape of OTs within specific regions. Moreover, while the majority of studies on the frequency of OTs have concentrated on demographic and clinicopathological data, our current investigation aims to delve deeper. Specifically, we illuminate the landscape of treatment options and provide insights into the follow-up information regarding OTs.

Three primary studies investigated the frequency of OTs in the Gulf region, covering Kuwait, Saudi Arabia, and the United Arab Emirates (Table 7) [8,9,10]. However, comparing our series to these studies presents challenges due to variations in time periods and sample sizes. Another complicating factor is the evolution of OT classification over time and the identification of new entities, such as POT [2]. Only Alsheddi M et al. provided the mean age of their cohort (29 years old), which closely aligns with the mean age of our cohort (30 years old) [10]. However, the mean age was not mentioned in the other two studies, making it difficult to extract this information from their data [8,9].

Nonetheless, OTs were consistently prevalent among males and in the mandibular site across all studies. Additionally, AM was the most common OT in Kuwait, and a previous study in Riyadh mirrored our findings [8,10]. Conversely, OD was found to be the most common OT in the United Arab Emirates [9]. It is worth mentioning that the paper by Al-Rawi N et al. [9] from the United Arab Emirates reported only AM and OD as OTs occurring in the maxillofacial regions in their series, raising the possibility that they did not capture all OTs present in their archive. None of the papers reported POT, which is a rare entity only described in 2014. Similar to our study, malignant OTs were rare, with only Alsheddi M et al. [10] reporting two cases (ameloblastic carcinoma and clear cell odontogenic carcinoma). The other two studies did not report any malignancies of odontogenic origin [8,9].

The mean age of patients diagnosed with OTs in the present study was 30, aligning with the mean age of the OT series reported from Tokyo, Japan; Nagpur, India; Cairo, Egypt; and a multicenter study in Nigeria [3,14,15,16]. However, certain studies have reported notably higher mean ages, such as Mascitti M et al. [17] from the Marche region, Italy, and Chrysomali E et al. [18] from Athens, Greece. OTs in the Riyadh population were more common in males, like most papers on the frequency of OT [3,14,16,17,18,19]. However, two different papers from Brazil reported a slight female predominance [20,21]. Conversely, a balanced male-to-female ratio was reported in some regions, such as Cairo, Egypt [15]. Mandible predilection was consistent with all published data [3,14,15,16,17,18,19,20,21,22].

In our series, AM accounted for more than half of the cases (63.0%); this observation was similar in Italy, Brazil, and India [14,17,21]. However, AM is still the most common OT in other regions with variable frequency (13.5% to 80.1%) [11]. Other studies found OD to be the most common OT [3,22,23] and the second most common OT in our series. OM was consistently the third most common OT in most OT series [11]. It is expected to find no POT records in the OT series published before the description of POT in 2014. Interestingly, POT has still not been reported in recent papers, emphasizing the rarity and lack of awareness of such an entity [3,23,24]. We speculate that a few POTs in some of the series are reported under OM [25,26]. Additionally, odontogenic malignancy appears rare across regions worldwide, ranging from 0.2% to 4.0% [3,14,15,16,17,18,20,21,22]. Our data are consistent with the reported percentage, with only 1.1% of the current series being malignant OT. However, Kebede et al. [27] reported a higher rate of 19.6%. The variation in frequency may be attributed to the lack of well-established diagnostic criteria for odontogenic malignancy. Hence, there is a need for stringent diagnostic protocols to reduce the frequency range and ensure accurate diagnosis (Table 8).

AM is a benign odontogenic tumor with aggressive behavior and a high recurrence rate if treated conservatively [28]. In our study, bone resection was the most used treatment option for AM, accounting for 66.7% of cases. Other treatment modalities included enucleation and excision. The recurrence rate of AM in this study was approximately 16.0%, with no significant difference among treatment modalities used. This recurrence rate is consistent with the rates reported in pooled data from 20 studies, which found a recurrence rate of 20%. However, radical treatment showed a significantly lower recurrence of 8.0%, compared to conservative treatment options such as enucleation or curettage, which had a recurrence rate of 41% [29]. In our case series, we observed recurrences in one case of OM and one OD. OM has a recurrence rate of 13.0% regardless of treatment, while it increases to 19.0% if treated conservatively [30]. The OM case in our series was treated conservatively with excision, which confirms the need for a more radical treatment of OM. On the other hand, OD recurrence is rare and poorly documented in the literature. It is likely due to incomplete removal or the presence of another OT in the affected area.

Our retrospective study is subject to inherent limitations arising from its dependence on existing medical records. We faced challenges concerning incomplete data, leading to data attrition and compromises in data validity. While retrospective studies provide valuable insights into past events, treatment practices, and outcomes, their findings may lack generalizability and applicability to broader populations. Therefore, the data presented require careful interpretation when drawing conclusions.

## 5. Conclusions

Our research offers valuable information on the epidemiology, demographic features, treatment trends, and recurrence rates of OTs in Riyadh, Saudi Arabia, supported by data collected from three prominent tertiary hospitals. Our findings underscore the predominance of AM as the most common OT and highlight the frequent occurrence of OTs in the mandible. Moreover, our observations regarding treatment modalities, with bone resection being the most prevalent approach, contribute to understanding current clinical practices. The documented recurrence rate, particularly among ameloblastoma cases, emphasizes the importance of long-term follow-up and underscores the need for further research to optimize management strategies and improve patient outcomes. Importantly, to the best of our knowledge, this study represents the only comprehensive investigation on OTs in Saudi Arabia, highlighting the necessity for similar studies across different regions to gain a more thorough understanding of these conditions nationwide.

## Figures and Tables

**Table 1:** The 5th edition of the WHO classification of odontogenic tumors, 2022 [2].

	Abbreviation	Definition	Clinical Presentation	Radiographic Presentation
Epithelial Odontogenic Tumors				
Ameloblastoma	AM			
Conventional ameloblastoma	cAM	A locally invasive and benign odontogenic tumor, most commonly in the posterior mandible.	Painless, slowly increasing swelling in the jaw, seen in the 4th to 5th decade, often asymptomatic with potential facial asymmetry and noticeable in cases in larger lesions.	Unilocular or multilocular radiolucency located in the posterior mandible. The multilocular type exhibits a soap bubble or honeycomb appearance, and root resorption of the adjacent teeth is frequent.
Unicystic ameloblastoma	UniAM	A distinct cystic variant constituting up to 25% of intraosseous AM.	Painless swelling is typically observed in the 2nd to 3rd decade, associated with the impacted 3rd molar in the posterior mandible.	Unilocular radiolucent area surrounding the crown of an impacted third molar.
Extraosseous/peripheral ameloblastoma	PeriAM	A rare variant comprising 1% of Ams; it arises either from the remnants of dental lamina within the oral mucosa or the basal cells of the surface epithelium.	Painless sessile nodule of the gingiva, occurring in the 5th to 6th decade and located in the premolar–molar region of the mandible.	N/A
Adenoid ameloblastoma	AdenoAM	Rare epithelial OT characterized by cribriform growth pattern, duct-like structures, and an occasional dentinoid, displaying aggressive behavior with a 70% recurrence rate.	Asymptomatic swelling with no site predilection; it may exhibit occasional pain and paresthesia.	The unilocular or multilocular with internal calcifications, cortical perforations, or root resorption.
Metastasizing ameloblastoma	MetAM	A histologically benign AM exhibiting metastasis to distant organs, commonly in the lungs.	Variable presentation and pulmonary metastasis may include a dry cough, hemoptysis, or dyspnea.	Similar to cAM in the jawbone
Adenomatoid odontogenic tumor	AOT	Uncommon, encapsulated OT with indolent behavior.	Known as two-thirds of a tumor because 2/3 occur in the 2nd decade, 2/3 occur in females, and 2/3 are associated with an impacted maxillary canine.	Unilocular radiolucency with variable radiopaque flecks (resembling snowflakes) is typically observed around the crown of an unerupted tooth, commonly the canine.
Squamous odontogenic tumor	SOT	Uncommon benign OT with squamous differentiation.	Painless swelling in the 4th decade, occasionally seen lateral to the roots of the teeth. Multiple or peripheral SOT has been reported.	Triangular or semicircular corticated radiolucency along the teeth roots. Tooth displacement occasionally seen.
Calcifying epithelial odontogenic tumor (Pindborg tumor)	CEOT	Uncommon benign epithelial OT with amyloid deposition and calcifications.	An asymptomatic, slowly growing mass occurring in the 4th decade and commonly in the posterior mandible.	Unilocular or multilocular radiolucency with variable radiodensity. Half of the cases were associated with an impacted tooth.
Epithelial and Mesenchymal Odontogenic Tumors				
Odontoma	OD	Hamartomatous growth exhibits different dental hard and soft tissues in various development stages.	Asymptomatic, slowly growing mass typically observed in the 2nd and 3rd decade, located in the anterior maxilla (compound) or posterior mandible (complex).	Compound: several tooth-like structures of varying sizes and shapes with a radiolucent rim. Complex: a calcified mass exhibiting radiodensity akin to the tooth structure, surrounded by a radiolucent rim.
Ameloblastic fibroma	AF	Benign-mixed OT without hard tissue deposition.	Asymptomatic, slowly growing lesion was seen in the second decade, most commonly in the mandible.	Unilocular or multilocular and corticated radiolucent lesion, 80% associated with an unerupted tooth.
Dentinogenic ghost cell tumor	DGCT	Rare benign OT displaying locally aggressive behavior, characterized by the abundance of ghost cells and dentinoid deposition.	Asymptomatic, slowly increasing swelling identified in the 3rd to 5th decade, typically localized in the posterior region of either jaw.	Well-defined, unilocular or multilocular-mixed radiolucent lesion. Tooth displacement or resorption is occasionally seen.
Primordial odontogenic tumor	POT	Recently described mixed POT exhibiting primitive dental tissue with occasional hard tissue deposition.	Slowly growing lesion in the first two decades and always associated with an unerupted tooth, commonly the third molar.	Well-demarcated, unilocular, bilocular, or multilocular radiolucency associated with an unerupted tooth.
Mesenchymal Odontogenic Tumors				
Odontogenic fibroma	OF	Rare OT consists mainly of mature fibrous tissue and inactive odontogenic epithelium with a peripheral variant (the most common peripheral odontogenic tumor).	Asymptomatic, slowly growing lesion seen in the fourth decade occurring commonly in the anterior maxilla and posterior mandible. Anterior maxillary lesions may cause soft tissue depression or dimpling.	**Central OF:** Unilocular or multilocular well-defined radiolucency is often seen intimately around the roots of teeth.
Cementoblastoma	CB	Benign neoplasm of cementoblasts, representing less than 3% of all OTs.	Slowly increasing painful swelling associated with teeth roots, most commonly the mandibular first molar.	The tumor appears as a radiopaque mass fused to one or more tooth roots and is surrounded by a thin radiolucent rim and resorption of the associated root is common.
Cemento-ossifying fibroma	COF	OT is derived from mesenchymal stem cells with differentiation towards periodontal structures, such as bone and cementum-like material.	Asymptomatic bony expansion in the posterior mandible mostly in the 3rd and 4th decades.	Well-demarcated radiolucency with a sclerotic rim in the tooth-bearing area of the jaws, accompanied by variable radiopacities. Bowing of the inferior border of the mandible may be evident.
Odontogenic myxoma	OM	The most common mesenchymal OT is composed of mainly myxoid stroma and occasional inactive odontogenic epithelium.	Painless swelling of the posterior mandible seen in the 2nd and 3rd decades.	Unilocular multilocular “honeycomb” or “tennis racket” radiolucency with diffuse borders and teeth displacement or resorption.

N/A: not applicable.

**Table 2:** Summary of demographics in the three tertiary centers.

	All Three Centers	KFMC	KAMC	PSMMC	*p*-Value
Cases	92	40 (43.5%)	28 (30.4%)	24 (26.1%)	
Age median (range)	29 (5–83)	33 (6–76)	22 (5–83)	26 (7–56)	0.4020
**Gender**					
Male	52	23	17	12	
Female	40	17	11	12	
Male/female ratio	1.27:1	1.16:1	1.54:1	1:1	0.6031
Mandible/maxilla ratio	2.64:1	2.25:1	2.8:1	3:1	0.884

**Table 3:** Distribution of OT among three different tertiary hospitals.

	All Three Centers	KFMC	KAMC	PSMMC
Epithelial Odontogenic Tumors				
Ameloblastoma	58 (63.0%)	27 (46.6%)	12 (20.7%)	19 (32.7%)
Adenoid ameloblastoma	1 (1.1%)	1 (100%)	0	0
Adenomatoid odontogenic tumor	2 (2.2%)	1 (50%)	0	1 (50%)
Ameloblastic carcinoma	1 (1.1%)	0	0	1 (100%)
Mixed Epithelial–Mesenchymal Odontogenic Tumors				
Odontoma	18 (19.5%)	3 (16.7%)	12 (66.6%)	3 (16.7%)
Ameloblastic fibroma	3 (3.3%)	3 (100%)	0	0
Primordial odontogenic tumor	1 (1.1%)	1 (100%)	0	0
Mesenchymal Odontogenic Tumors				
Odontogenic myxoma	4 (4.3%)	3 (75.0%)	0	1 (25.0%)
Central odontogenic fibroma	2 (2.2%)	1 (50%)	1 (50%)	0
Cementoblastoma	2 (2.2%)	0	2 (100%)	0
Total	92 (100%)	40 (43.5%)	28 (30.4%)	24 (26.1%)

**Table 4:** Summary of the clinicopathological features of odontogenic tumors.

	Number of Case	Age Median (Range)	Gender		Location *	
			M	F	Mandible	Maxilla
Epithelial Odontogenic Tumors						
Ameloblastoma	58 (63.0%)	36 (6–83)	34 (58.6%)	24 (41.4%)	47 (81.0%)	11 (19.0%)
Conventional amelobastoma	54 (93.1%)	38 (6–83)	33 (61.1%)	21 (38.9%)	44 (81.5%)	10 (18.5%)
Unicystic ameloblastoma	4 (6.9%)	20 (16–51)	1 (25.0%)	3 (75.0%)	3 (75.0%)	1 (25.0%)
Adenoid ameloblastoma	1 (1.1%)	14 (N/A)	0	1 (100%)	0	1 (100%)
Adenomatoid odontogenic tumor	2 (2.2%)	17 (15–19)	1 (50.0%)	1 (50.0%)	1 (50.0%)	1 (50.0%)
Ameloblastic carcinoma	1 (1.1%)	31 (N/A)	1 (100%)	0	1 (100%)	0
Mixed Epithelial–Mesenchymal Odontogenic Tumors						
Odontoma	18 (19.5%)	20 (5–50)	10 (55.6%)	8 (44.4%)	8 (47.1%)	9 (52.9%)
Complex	10 (55.6%)	17 (5–50)	5 (50.0%)	5 (50.0%)	8 (80.0%)	2 (20.0%)
Compound	8 (44.4%)	17 (9–37)	5 (62.5%)	3 (37.5%)	0	7 (100%)
Ameloblastic fibroma	3 (3.3%)	13 (6–26)	1 (25.0%)	2 (27.5%)	2 (75.0%)	1 (25.0%)
Primordial odontogenic tumor	1 (1.1%)	16 (N/A)	1 (100%)	0	1 (100%)	0
Mesenchymal Odontogenic Tumors						
Odontogenic myxoma	4 (4.3%)	31 (27–36)	3 (75.0%)	1 (25.0%)	2 (50.0%)	2 (50.0%)
Central odontogenic fibroma	2 (2.2%)	30 (15–45)	1 (50.0%)	1 (50.0%)	2 (100%)	0
Cementoblastoma	2 (2.2%)	32 (17–48)	0	2 (100%)	2 (100%)	0
Total	92 (100%)		52 (56.5%)	40 (43.5%)	66 (72.5%)	25 (27.5%)

* The location of one case of odontoma was not reported.

**Table 5:** Summary of the treatment modalities of each odontogenic tumor.

	Number of Cases	Treatment		
		Enucleation	Excision	Resection
Epithelial Odontogenic Tumors				
Ameloblastoma	52 (63.4%)	6 (11.5%)	12 (23.1%)	34 (65.4%)
Conventional ameloblastoma	48 (92.3%)	4 (8.3%)	12 (25.0%)	32 (66.7%)
Unicystic ameloblastoma	4 (7.7%)	2 (50.0%)	0	2 (50%)
Adenoid ameloblastoma	1 (1.2%)	0	0	1 (100%)
Adenomatoid odontogenic tumor	1 (1.2%)	1 (100%)	0	0
Ameloblastic carcinoma	1 (1.2%)	0	0	1 (100%)
Mixed Epithelial–Mesenchymal Odontogenic Tumors				
Odontoma	17 (20.8%)	12 (70.6%)	4 (23.5%)	1 (5.9%)
Ameloblastic fibroma	2 (2.4%)	1 (50.0%)		1 (50.0%)
Primordial odontogenic tumor	1 (1.2%)	0	0	1 (100%)
Mesenchymal Odontogenic Tumors				
Odontogenic myxoma	4 (5.0%)	0	1 (25.0%)	3 (75.0%)
Central odontogenic fibroma	1 (1.2%)	1 (100%)	0	0
Cementoblastoma	2 (2.4%)	2 (100%)	0	0
Total	82 (100%)	23 (25.6%)	17 (21.0%)	42 (51.0%)

**Table 6:** Summary of the follow-up information for odontogenic tumors that showed recurrence.

	Number of Cases	Recurrence	No Recurrence	Follow-Up Period
Ameloblastoma	50	8 (16.0%)	42 (84.0%)	1 year–6 years
Odontoma	17	1 (5.9%)	16 (94.1%)	1 year–3 years
Odontogenic myxoma	4	1 (25.0%)	3 (75.0%)	7 months–4 years
Ameloblastic fibroma	2	1 (50.0%) *	1 (50.0%)	1 year
**Total**	73	11 (15.1%)	62 (84.9%)	N/A

* Recurrence after one year of ameloblastic fibrosarcoma and succumbing to the disease due to liver and lung metastasis after six years.

**Table 7:** Comparison of OT distribution in the current study and other Saudi and Gulf countries.

	Current Study (Three Centers), Riyadh, SA	Ali MA et al., Kuwait University, Jabriya, Kuwait [8]	Alsheddi M et al., King Saud University, Riyadh, KSA [10]	Al-Rawi N et al., Tawam Hospital, Abu Dhabi, UAE [9]
Sample size	92	27	108 *	22
Period	11 years	6 years	26 years	20 years
Mean age	30	N/R	29	N/R
Male/female ratio	1.3:1	1.25:1	1.4:1	1:1
Mandible/maxilla ratio	2.64:1	3.5:1	2.1:1	1.62:1
Epithelial Odontogenic Tumors				
Ameloblastoma	58 (63.0%)	17 (63.0%)	47 (43.5%)	4 (18.1%)
Adenoid ameloblastoma	1 (1.1%)	N/R	N/R	N/R
Adenomatoid odontogenic tumor	2 (2.2%)	N/R	8 (7.4%)	N/R
Calcifying epithelial odontogenic tumor	N/R	1 (3.7%)	2 (1.8%)	N/R
Ameloblastic carcinoma	1 (1.1%)	N/R	1 (0.9%)	0 (0%)
Clear cell odontogenic carcinoma	N/R	N/R	1 (0.9%)	N/R
Mixed Epithelial–Mesenchymal Odontogenic Tumors				
Odontoma	18 (19.5%)	9 (33.3%)	28 (26.0%)	17 (77.8%)
Ameloblastic fibroma	3 (3.3%)	N/R	4 (3.7%)	N/R
Primordial odontogenic tumor	1 (1.1%)	N/R	N/R	N/R
Dentinogenic ghost cell tumor	N/R	N/R	N/R	N/R
Mesenchymal Odontogenic Tumors				
Odontogenic myxoma	4 (4.3%)	N/R	12 (11.1%)	N/R
Central odontogenic fibroma	2 (2.2%)	N/R	1 (0.9%)	N/R
Cementoblastoma	2 (2.2%)	N/R	4 (3.7%)	N/R
Total	92 (100%)	27 (100%)	108 (100%)	22 (100%)

* The total number of cases reported in this series was 188, but we excluded keratocystic odontogenic tumors and calcifying cystic odontogenic tumors since they are reclassified as cysts. N/R; not reported.

**Table 8:** Comparison of OT distribution in the current study and selected international studies.

	Current Study (Three Centers), Riyadh, SA	Kokubun K et al. Tokyo, Japan [3]	de Medeiros WK et al. Natal, Northeastern Brazil [20]	Mascitti M et al. Ancona, Italy [17]
Sample size	92	1089	247	100
Period	11 years	45 years	22 years	25 years
Mean age	30	29	28	49.7
Male/female ratio	1.3:1	1.2:1	1:1.2	1.78:1
Mandible/maxilla ratio	2.64:1	2.1:1	2:1	2.1:1
Epithelial Odontogenic Tumors				
Ameloblastoma	58 (63.0%)	456 (41.9%)	112 (45.4%)	56 (56%)
Adenoid ameloblastoma	1 (1.1%)	N/R	N/R	N/R
Adenomatoid odontogenic tumor	2 (2.2%)	17 (1.6%)	10 (4.0%)	2 (2.0%)
Squamous odontogenic tumor	N/R	2 (0.2%)	N/R	1 (1.0%)
Calcifying epithelial odontogenic tumor	N/R	8 (1.6%)	5 (2.0%)	4 (4.0%)
Ameloblastic carcinoma	1 (1.1%)	1 (0.1)	1 (0.4%)	2 (2.0%)
Primary intra-osseous carcinoma	N/R	8 (0.7%)	N/R	N/R
Clear cell odontogenic carcinoma	N/R	N/R	1 (0.4%)	1 (1.0%)
Mixed Epithelial–Mesenchymal Odontogenic Tumors				
Odontoma	18 (19.5%)	463 (42.5%)	89 (36.1%)	17 (17.0%)
Ameloblastic fibroma	3 (3.3%)	17 (1.6%)	4 (1.6%)	3 (3.0%)
Primordial odontogenic tumor	1 (1.1%)	N/R	N/R	N/R
Dentinogenic ghost cell tumor	N/R	7 (0.6%)	1 (0.4%)	2 (2.0%)
Mesenchymal Odontogenic Tumors				
Odontogenic myxoma	4 (4.3%)	41 (3.8%)	17 (6.9%)	4 (4.0%)
Central odontogenic fibroma	2 (2.2%)	22 (2.0%)	3 (1.2%)	1 (1.0%)
Cementoblastoma	2 (2.2%)	8 (0.7%)	4 (1.6%)	2 (2.0%)
Cemento-ossifying fibroma	N/R	38 (3.5%)	N/R	4 (4.0%)
Odontogenic sarcoma	N/R	1 (0.1%)	N/R	1 (1.0%)
Total	92 (100%)	1089 (100%)	247 (100%)	100 (100%)

N/R; not reported.

## Data Availability

The data presented in this study are available on request from the corresponding author. The data are not publicly available due to the hospital policies.
